# Non-Traditional Management of the Neurogenic Bladder: Tissue Engineering and Neuromodulation

**DOI:** 10.1100/tsw.2007.178

**Published:** 2007-08-17

**Authors:** Jane M. Lewis, Earl Y. Cheng

**Affiliations:** Children's Memorial Hospital, Feinberg School of Medicine Northwestern University, Chicago, IL, USA

**Keywords:** tissue engineering, bladder reconstruction, neuromodulation, bladder stimulation, neurogenic bladder

## Abstract

Patients with spina bifida and a neurogenic bladder have traditionally been managed with clean intermittent catheterization and pharmacotherapy in order to treat abnormal bladder wall dynamics, protect the upper urinary tract from damage, and achieve urinary continence. However, some patients will fail this therapy and require surgical reconstruction in the form of bladder augmentation surgery using reconfigured intestine or stomach to increase the bladder capacity while reducing the internal storage pressure. Despite functional success of bladder augmentation in achieving a low pressure reservoir, there are several associated complications of this operation and patients do not have the ability to volitionally void. For these reasons, alternative treatments have been sought. Two exciting alternative approaches that are currently being investigated are tissue engineering and neuromodulation. Tissue engineering aims to create new bladder tissue for replacement purposes with both “seeded” and “unseeded” technology. Advances in the fields of nanotechnology and stem cell biology have further enhanced these tissue engineering technologies. Neuromodulation therapies directly address the root of the problem in patients with spina bifida and a neurogenic bladder, namely the abnormal relationship between the nerves and the bladder wall. These therapies include transurethral bladder electrostimulation, sacral neuromodulation, and neurosurgical techniques such as selective sacral rhizotomy and artificial somatic-autonomic reflex pathway construction. This review will discuss both tissue engineering techniques and neuromodulation therapies in more detail including rationale, experimental data, current status of clinical application, and future direction.

## INTRODUCTION

When patients with spina bifida and a neurogenic bladder store urine under high pressure, upper tract deterioration and renal failure can occur necessitating the need for surgical reconstruction of the bladder.

The advancements in surgical reconstruction of the genitourinary tract have been significant over the past half-century. Successful augmentation of the neurogenic bladder with a piece of reconfigured intestine (enterocystoplasty) or stomach (gastrocystoplasty) has been previously demonstrated[1]. Although much has been learned from the experience with bladder augmentation in terms of what can be done technically better in the operating room, it is generally accepted that while bladder augmentation provides the patient with an adequate urinary reservoir, this functional improvement is not risk-free and is associated with significant complications. Some of the complications of conventional enterocystoplasty include: infection, stone formation, intestinal obstruction, electrolyte imbalances, metabolic disturbances, bladder perforation, and possible tumor development. These potential complications demonstrate the need to investigate other management strategies for the neurogenic bladder found in patients with spina bifida.

Two strategies currently under investigation that hold great promise for neurogenic bladder management are tissue engineering and neuromodulation. Tissue engineering aims to regenerate new bladder tissue for augmentation purposes while neuromodulation attempts to alter the innervation of the neurogenic bladder to allow it to function in a more normal fashion. Unlike traditional surgical therapy, neuromodulation therapy directly addresses the root of the problem of a neurogenic bladder; i.e. the abnormal innervation that results in a dysfunctional bladder due to the ongoing bad relationship between nerves and the detrusor layer of the bladder. This article will review the current status of tissue engineering and neuromodulation as it relates to the neurogenic bladder.

## TISSUE-ENGINEERING FOR BLADDER RECONSTRUCTION

The need for new tissue to perform surgical reconstructions, in many organ systems, has driven forward the field of tissue engineering. Two basic technologies have been used in tissue engineering: “unseeded” and “seeded.” Unseeded technology involves the incorporation of a scaffold material (synthetic or biologic) into the host organ, which is used as a template for the ingrowth of native cells that then initiate the regenerative process. Seeded technology is similar to unseeded, but with the addition of cultured cells to the scaffold prior to implantation into the host. Initial successes in animal models have been achieved with both unseeded and seeded technologies. Current advances in the areas of nanotechnology and stem cell biology aim to build on these initial successes to improve the degree of bladder regeneration that can be achieved.

### Unseeded Technology

Intuitively, for over a century, surgeons have looked to various replacement alternatives when the native bladder is no longer adequate[2]. Early attempts to augment the bladder in an “unseeded” fashion involved the use of synthetic materials including silicone, rubber, polytetrafluoroethylene, and polypropylene.These synthetic and non-biodegradable materials failed due to host–foreign body reactions[3,4,5,6,7]. Grafts became encrusted, infected, and were inappropriate for clinical use[8]. This unfavorable experience with synthetic materials illuminated the need to develop a graft material that would be minimally immunogenic, ideally biodegradable, and one that would allow in-growth of native cells for successful tissue regeneration. Functionally, the graft should promote organogenesis of new bladder tissue that provides a low-pressure reservoir for protection of the upper urinary tract and healthy new tissue that will be stable long term without graft contraction. This led to the use of biodegradable materials, such as placenta, amnion, skin, peritoneum, omentum, dura and pericardium. Animal studies have demonstrated that most of these biodegradable materials result in variable degrees of bladder regeneration, can increase bladder capacity, and have less complications as compared to those that have been seen with synthetic grafts[9,10,11,12]. Despite these initial encouraging results in animals, there are no reports of human clinical use. Presumably, problems were encountered long-term with graft contraction and loss of capacity that limited the ability to proceed with clinical trials.

More recently, new non-synthetic, biodegradable materials have been developed from different organs other than the placenta, amnion and pericardium. Specifically, the bladder, stomach, and small intestine have all been utilized. Each of these organs are harvested from a sacrificed animal and then processed with detergents and mechanical shearing to render the organ fragment “acellular.” The remaining tissue is referred to as an “acellular extracellular matrix” (ECM), and can be used as an unseeded graft for bladder augmentation purposes[13,14,15].

The most thoroughly studied collagen-based ECM graft is small intestinal submucosa (SIS)[16]. SIS is derived from pig small intestine. Using mechanical force, the mucosal layer is removed from the inner surface and the serosa and muscularis layers are removed from the outer surface. The result is a thin, translucent membrane (0.1 mm wall thickness) composed mainly of the submucosal layer of the intestinal wall. Even though SIS is acellular, it has been shown to be biologically active. It contains a unique combination of intrinsic growth factors, cytokines, structural proteins, glycoproteins, and proteoglycans. These factors likely aid in the regenerative process, including cell migration, cell-to-cell interaction, and cell growth and differentiation[17,18,19]. A potential concern for any biologic material derived from another host is its immunogenic properties. SIS has been tested directly in over 1000 cross-species transplants with no significant immune system response[20,21,22]. The combined biologic properties of SIS make it very attractive for use in an unseeded fashion for tissue regeneration.

Initially, SIS was utilized in animal studies as a vascular patch, where tissue-specific regeneration was rapidly observed in arteries and veins[23,24,25]. Soon afterward, its use was tested in bladders, using both rat and canine animal models[26]. Not only did the resultant bladder tissue at harvest show all three layers of bladder wall (urothelium, smooth muscle, and serosa), but the augmented bladders were found to be compliant, with good capacity and functionally innervated as shown by urodynamic studies performed up to 15 months after the augmentation[27]. After sacrifice, *in vitro* muscle strip studies comparing normal bladder as well as regenerated portions of the bladder showed similar contractility and compliance[28]. An important observation in these early studies utilizing SIS as a bladder augmentation material and subsequent studies in large animal models is that the degree of bladder regeneration achieved with different pieces of SIS can be very variable and somewhat unpredictable. This, in part, relates to the fact that SIS is a biologic material whose activity can vary depending on the source of the material. Studies looking at different forms of SIS have demonstrated that grafts manufactured from the distal ileum provide more reliable bladder regeneration than a segments harvested from the same animal’s proximal jejunum[29,30]. How the factors vary between the ileum-derived SIS and the jejunum-derived SIS, producing different forms of regeneration, is unknown. This lack of understanding of the important factors involved in promoting successful bladder regeneration emphasizes the need for a better understanding of the specific cellular mechanisms involved in this process. While there have been partial mechanistic reports on various biomaterials[31,32], as well as work done on the cell-to-cell interactions and the intrinsic growth factors[33,34,35,36] much more work is still necessary. Once we better understand how native cells from the bladder interact with an ECM to initiate the regenerative process and the cellular factors that promote successful organogenesis, then we will be able to better understand why there are variable responses to various ECMs and enhance our ability to develop the best ECMs for bladder regeneration.

The initial successful results with SIS in animal models decribed above involved replacement of 40% of the animal bladder. When SIS was used in subtotal cystectomy models in which 90% of the bladder was removed, the results were uniformly poor. The SIS grafts showed dense adhesions, graft shrinkage, and calcification at the graft site[37]. Histologic evaluation demonstrated that there were areas of successful regeneration at the periphery of the graft suggesting the reason for failure was a lack of nutrition and neovascularization of the central portion of the graft.

Other ECMs similar to SIS have also been investigated as a biodegradabale graft for bladder augmentation. Homologous bladder acellular matrix grafts (BAMG) have been shown to be successful in rats, demonstrating morphologic as well as functional regeneration[33,38]. Also, encouraging results were observed when bladder acellular matrix allografts (BAMA) were used in a porcine model. Specifically, multilayered bladder growth was seen at 30 days, including urothelium, angiogenesis, and organized smooth muscle regeneration[15]. However, similar to SIS, long term results in large animal models have shown eventual graft shrinkage which likely relates to lack of adequate neovascualrization[39].

In summary, SIS and other similar ECMs have demonstrated that successful bladder regeneration can be achieved when these grafts are used in an unseeded fashion for bladder augmentation. While these findings are very promising, there are still several concerns, which keep this present technology from being used clinically in humans. First, the use of large grafts has been unsuccessful, hence limiting the possibility of total or sub-total bladder replacement[37]. Efforts to enhance neovascularization of the graft to allow for success with large grafts are under investigation. Second, consistent manufacturing of these grafts with predictable biologic activity has not yet been achieved. When the mechanism of action as to what factors promote ingrowth, what will promote neovascularization, etc. is better elucidated, we will hopefully have the ability to provide a more uniform biodegradable graft which uniformly promotes successful bladder regeneration.

## Seeded Technology

Another approach to tissue engineering involves “seeded” technology. This involves the use of cells derived from a small piece of autologous tissue, which is harvested from the patient. These cells are established in primary culture in the laboratory, expanded rapidly, and then seeded *in vitro* on a scaffold prior to placement into the bladder. Theoretically, this approach could have advantages over the unseeded approach in that it could “jump start” the regenerative process and not entirely rely on ingrowth of cells from the native bladder once the graft is placed *in situ*. In order for this approach to be successful, one needed to demonstrate protocols that could reliably separate bladder smooth muscle cells and urothelial cells and then establish them in culture with subsequent expansion. One initial challenge was the *in vitro* growth of urothelial cells, which were initially thought to have a preprogrammed death. Eventually, several protocols were developed for keeping *in vitro* urothelial cells alive were developed and refined[40,41,42,43]. Additional refinements in technique have resulted in the ability to establish primary cultured bladder cells that could then be expanded to cover a surface area of 4200 $m^2$ within 8 weeks[40].

Once the cells are grown *in vitro*, they are seeded on a biodegradable membrane and then transplanted back into the host for continuation of cell growth and differentiation. The choice of biodegradable membrane for seeded technology is a point of ongoing research and development. As discussed in the section on unseeded technology, the goal of the graft is to support cell growth, while not causing an immune response, to biodegrade with time, and to be easily reproducible. The material that has been studied most extensively in the setting of seeded technology for bladder regeneration is a synthetic biodegradable polymer sheet of non-woven polyglycolic acid. This comes from the chemical family of poly-$\alpha$-esters: poly-L-lactic acid (PLLA), polyglycolic acid (PGA), and their copolymers (PLGA). These degrade through hydrolysis to form standard byproducts of cell respiration, and are well known for surgical applications; e.g. as Vicryl sutures. These polymers are easily processed into highly porous fibrous meshes, which can support cell ingrowth and proliferation.

PGA was initially shown to allow the successful organized ingrowth of rabbit and human bladder cells (both urothelial and smooth muscle cells) when these cells were seeded *in vitro* onto the PGA scaffold[44]. This polymer was then tested as an augment material in dog bladder, after being seeded with urothelium and smooth muscle cells, and was found to develop in an organized fashion in the dog bladder. Successful regeneration of bladder tissue, which was histologically similar to native bladder tissue, was achieved. Significant increase in bladder capacity and improvement in compliance was noted. When seeded grafts were compared to unseeded grafts, greater increases in bladder capacity and less graft contraction were observed in the seeded grafts. This suggested that the seeded cells resulted in an enhanced regenerative response although the mechanism in how these cells promoted this response remains to be elucidated[45,46].

These encouraging results in the use of seeded grafts in animals have led to a recent trial in humans. Bladder augmentation using PGA composite grafts, in a seeded fashion, was carried out in seven patients with spina bifida and a neurogenic bladder[47]. All of the patients survived the operation. Although there was histologic evidence of some degree of bladder regeneration in these patients, there was a lack of uniform successful increase in bladder capacity and improvement in bladder wall compliance as compared to that routinely achieved with conventional enterocystoplasty. Patients still were not able to spontaneously empty and required intermittent catheterization. While these results are landmark in nature in that they demonstrate that current tissue engineering technology represents translational research that can be applied successfully clinically, the lack of adequate augmentation of the bladder in these patients indicates that the technology still requires improvement and is not ready for widespread clinical application.

## Nanotechnology

The advantage of a biomaterial like SIS is that it has inherent biologic activity that is important for regeneration but a major disadvantage is the variability present in this biologic activity. The advantage of synthetic materials like PGA is its uniformity but its major disadvantage is precisely its absence of biologic activity. Recent advancements in nanotechnology may allow us to utilize the advantages of both types of material to develop synthetic biomaterials that have predictable biologic activity. The field of molecular self-assembly has recently made significant strides towards applications in regenerative medicine[48]. The concept is to intentionally engineer materials in the organization observed throughout nature; e.g. non-covalent assembly of phospholipids into bilayers, vesicles, and micelles. An example of this is the use of peptide-amphiphiles molecules that are triggered to self-assemble when placed in aqueous solution, or by changing the pH or ionic gradient[49]. When this technology has been applied to bladder tissue engineering, significant effects on bladder smooth muscle growth on nanodesigned grafts were noted[50]. Current work by several laboratories is being directed at nanodesigning synthetic materials that can be integrated with important growth factors known to facilitate bladder regeneration and neovascularization. This technology is quite promising for the delivery of intrinsic factors found to be necessary for support of both seeded cells and native cells in the bladder that invades the graft.

## Stem Cells

Another limiting factor specific to both unseeded and seeded technology is whether bladder cells harvested from a neurogenic bladder continue to be dysfunctional and “neurogenic” in their behavior or whether they behave in a normal fashion when they are involved in the regenerative process. If the cells from the neurogenic bladder do not have the capacity to convert to a normal phenotype, then the resultant regenerated bladder tissue from either unseeded or seeded techniques will not result in normal bladder tissue, which is not the desired result. When smooth muscle cells from the neurogenic bladder have been studied *in vitro*, they have been shown to retain significant differences when compared to normal cells[51,52]. Ongoing research is investigating these cellular differences. However, these findings raise concern that we may need to look at alternative cell sources for bladder regeneration. The recent explosion of stem cell research for regenerative medicine has fueled recent studies investigating the utility of stem cells for use in bladder regeneration. It is important to note that the majority of work that has been done in this area has been with the use of peripheral stem cells that are not totipotential in their ability to make an embryo, which is different from embryonic stem cells that carry with them the ethical concerns of their appropriate use.

The concept is elegant: harvest peripheral stem cells from a patient, possibly by taking a bone marrow biopsy, then place these cells into *in vitro* environments (e.g. culture and surface tension conditions) to cue their differentiation into the tissues that make up the bladder. Therefore, there are no immunogenic concerns, and theoretically since the new bladder tissue has not had development exposure to the abnormal nervous system innervation, this bladder tissue would be non-neurogenic. Stem cell regenerative technology has been used in a variety of urological structures[53]. Specific to bladder regeneration, successes in smooth muscle differentiation from bone-marrow derived mesenchymal stem cells has been shown in augmented rat bladders in an experiment comparing unseeded SIS to stem cell seeded SIS. Most notable was the decreased time to demonstration of multiple bladder tissue layers[54].

Clearly, bladder regeneration is possible using both unseeded and seeded tissue-engineering technologies. These are exciting times in that numerous investigators are making rapid new advancements in the field of bladder tissue engineering. Further advances in current techniques will eventually revolutionize urologic reconstructive surgery, as we know it today. However, it is extremely important to note that enterocystoplasty is still the gold standard for augmentation of the neurogenic bladder and that current tissue engineering technologies are not ready for wide spread clinical application. Nevertheless, it is the hope of all that work in this field of regenerative medicine that conventional bladder augmentation with bowel segments will be more of an historical footnote with primary therapy of the neurogenic bladder directed at tissue engineering techniques to promote normal organogenesis.

## NEUROMODULATION

Neuromodulation therapy aims to treat the root of the problem of a neurogenic bladder; namely the abnormal innervation of the bladder. Different forms of this therapy are centered on trying to retrain the nerve-muscle interaction to attain more normal bladder function. This type of therapy includes a spectrum: non-operative measures such as transurethral electrical bladder stimulation, minimally invasive procedures such as implantation of a sacral neuromodulation pacemaker device, as well as operative measures that reconfigure sacral nerve root anatomy. All three of these neuromodulation therapies will be now discussed in further detail.

### Transurethral Bladder Stimulation

The use of intravesical transurethral bladder stimulation dates back to the 1950’s in Hungary. This initial group of investigators described their experience with their first 100 patients[77]. Spontaneous voiding along with acceptable continence was observed in up to 70% of their patients. While the exact mechanism of action at that time was not understood (and to this day remains poorly understood), subsequent experiments in feline and rat bladders have led researchers to believe that electrical stimulation of the afferent mechanoreceptors causes a reflexive activation of the detrusor muscle[55]. Since the 1970’s, other centers around the world have attempted to apply this treatment strategy to different forms of neurogenic bladder. The next published study using this technique involved patients who had suffered an incomplete spinal cord lesion. Nearly 60% of a small cohort of patients developed “perfect bladder control” and 93% could be considered socially continent[56]. Given these intial encouraging results, this method for addressing neurogenic bladder dysfunction was applied to the pediatric spina bifida population in the United States starting in the 1980’s[57].

The technique for bladder stimulation, though non-surgical, is labor intensive. All patients must undergo an initial urodynamic study consisting of a cystometrogram (CMG) at a slow-fill rate where leak-point pressure is measured at the first sign of urethral meatal leakage. At bladder capacity, the corresponding detrusor pressure is used to calculate the bladder compliance. The bladder is emptied and this volume is recorded as the official bladder capacity. This is followed by a 15-minute bladder observation period with the bladder filled to half-capacity with normal saline via an electrocatheter. The first actual treatment session follows, which is a 90 minute session where various parameters, such as intensity of current, frequency, impulse duration, time to arrive gradually at the maximum current, the time spent at maximum current (together known as package time), interval time between packages, can be adjusted for the individual patient. In general, the hypotonic bladder will receive a stronger and more frequent package than a hypertonic bladder. A series of treatment consists of 20 distinct 90-minute sessions. Follow-up CMG is performed 3-6 months later, and follow-up stimulation sessions are tailored to each individual patient's needs.

Overall, the clinical results of intravesical transurethral bladder stimulation in patients with spina bifida have been variable. In early preliminary series, 38% of patients were able to achieve satisfactory urinary continence and void with low pressures[57,58]. Other trials at separate institutions were not as encouraging. Improved continence was not observed and sustained improvements in bladder capacity were noted in only half of the treated patients[59,60]. Additional preliminary trials at various centers found no significant differences between treatment and control groups and discontinued the practice[61]. Later, a larger scale, multi-center trial was undertaken where 335 patients were treated with bladder stimulation therapy with a uniform protocol. Following treatment, 53% of patients increased their bladder capacity by more than 20%, 37% of patients showed no change in capacity, and 10% showed a 20% decrease. Of the "nonresponder" patients in which a decreased in bladder capacity was noted, their capacity at the initiation of therapy was much closer to age-expected than those who were "responders" (83% versus 65% age-expected bladder capacity). This suggests that the larger, more atonic, bladders may be more resistant to this form of therapy. On the other hand, of the more than half of patients who were "responders", their bladder capacity increase did not occur at the expense of an increase in bladder pressure: 16% of patients had improved bladder wall compliance at bladder capacity, and 74% showed no change in compliance. The average bladder pressure at capacity was less than 30 cm $H_2O$ for these two groups combined[62].

Bladder stimulation therapy has also been found to be effective in patients with a "high risk" neurogenic bladder—those patients with pre-treatment bladders <60% of expected bladder capacity and detrusor pressure at capacity of > 50 cm $H_2O$. In a preliminary series, 7 patients with high-risk urodynamic findings that were planned for surgical intervention in the form bladder augmentation were treated with bladder stimulation prior to proceeding to surgery. Four of the 7 patients were noted to have a significant increase in bladder capacity and improvement in compliance while the remaining three patients had modest improvements. Following treatment, none of the 7 patients went on to require bladder augmentation[63].

From the greater than 25 years of experience with intravesical transurethral bladder stimulation, several conclusions can now be made. In general, bladder stimulation therapy will provide volitional voiding in less than 10% of patients. It has greater effects on the detrusor muscle than it does on the sphincter muscle (bladder outlet). It can be an effective form of treatment for up to 60% of patients in which significant increases in bladder capacity and improved compliance can be expected. In patients that respond to therapy in a positive fashion, the beneficial effects are generally permanent. The therapy is very labor intensive and requires a very dedicated family. However, when effective it can improve bladder wall dynamics to the point where it may decrease the need for medications and avoid the need for augmentation. Lastly, it appears that bladder stimulation is more effective in younger patients suggesting that older patients have less reversible changes in the bladder. A better understanding of the mechanism of action will hopefully lead to more effective forms of bladder stimulation therapy in the future.

## Sacral Neuromodulation—"InterStim"

Another, at this point experimental, technique for management of neurogenic bladder dysfunction is sacral neuromodulation, also known by its trade name "InterStim." This is a reversible, implantable device that was approved for use in the United States in 1997 for adults suffering from severe urgency and urge incontinence. The device is implanted, in adults, under local or general anesthesia, with the patient lying prone and with the knee and pelvis positioned so that the sacrum is as horizontal as possible. In the first phase of placement, an insulated spinal needle is used to percutaneously access the sacral foramen, which will stimulate the S3 nerve root. The needle is electrically stimulated and repositioned until anal contraction and/or plantar flexion of the great toe is observed. Usually, both sides of the sacrum are tested to determine which side gives the best response. The neurostimulator lead is then placed through a wider, hollow needle placed alongside the initial finder needle and this is retested for placement. This lead is tunneled under the skin to provide adequate distance subcutaneously between the surface of the skin and the nerve roots. This lead is then attached to a temporary, external test stimulator that can be adjusted to deliver different levels of current. If after a test period of 1 week the patient experiences amelioration of symptoms, then a permanent pacemaker device is implanted, usually in the area of the buttocks, and is attached to the neurostimulator lead. If no improvement is noted, the lead is removed.

The rationale for the development of this device comes from the same logic as intravesical transurethral bladder stimulation[77]. While the exact mechanism of action of both of these types of neuromodulation is not completely understood, two concepts are generally accepted as to its efficacy. One is that the on-going stimulation of the efferent fibers of the sacral nerve root decreases parasympathetic outflow to the bladder, hence reducing bladder instability. The other is that the rhythmic contractions of the pelvic floor leads to more coordinated micturition by engaging existing reflex pathways[64]. A theory as to how this is achieved is that the stimulation of myelinated A delta fibers, especially from the sacral nerve roots, inhibits the detrusor muscle by increasing the tone of the sphincter and pelvic floor[65,66].

While this technique of neuromodulation has been used to treat voiding dysfunction in adults, the first report of use in children was not until recently when it was reported to improve symptoms of detrusor overactivity in 76% of the 41 urge syndrome patients treated[67]. A group in Italy tried percutaneously stimulating the tibial nerve and observed improvements in their patients with overactive bladder or with urinary retention, both by symptom report and by urodynamics[68]. Sacral nerve stimulation was also attempted in neurologically “normal” children diagnosed with dysfunctional elimination syndrome (DES), which is a constellation of functional genitourinary and gastrointestinal symptoms. In a series of 23 children with DES who had not improved after a motivated 6 months of medical therapy, 84% of patients had resolution or improvement of incontinence and 69% had resolution or improvement of nocturnal enuresis at 13 months follow-up. The majority of the patients experienced improvement in pain, urgency, frequency, and constipation, as well as a reduction in the amount of medication required[69].

The only published study to date involving sacral neuromodulation in patients with neurogenic bladder due to spina bifida comes from France. The study was carried out in a prospective, randomized fashion with 26 boys and 16 girls, average age 11.9 years, assigning 21 patients to treatment and 21 patients to a control group. Incontinence and urodynamic outcomes were compared. The implanted group had a significantly improved leak point pressure, but the control group actually had the significantly improved bladder capacity at 12 months, despite the treatment group appearing better at the 6 and 9 month intervals. The treatment group did have 9 patients report improvement in intestinal transit time, 5 report a disappearance of urinary tract infections, and 6 report a persistent sensation of a full bladder. No such improvements were noted in the control group[70]. This pilot study certainly warrants further investigation into this therapy’s promising use in the spina bifida population.

## Neurosurgery Options—Selective Sacral Rhizotomy and Artificial Somatic-Autonomic Reflex Pathway

As with bladder stimulation and sacral neuromodulation, all of the neurosurgical options are based on an increased understanding of neurourology, which has been growing over the past 50 years. Given the observations of improved bladder function in children with cerebral palsy and myelomeningocele who underwent selective sacral rhizotomy to improve their lower limb spasticity[71,72], this surgical technique has been applied specifically for patients that desire an improvement in bladder function, indepenedent of their neuromuscular function. The patients that have been referred for this very invasive neurosurgical method of treatment have failed medical management. Sacral rhizotomy is accomplished by careful surgical exposure of the sacral nerve roots, stimulation of these nerves and transection of those nerves which produce a definitive rise in detrusor pressure (as determined by intraoperative urodynamic monitoring) and no simultaneous contraction of the external sphincter. In a preliminary series of 11 patients followed for up to 4 years, there was a 93% increase in bladder volume at a pressure of 40 $cmH_20$. All had improvement in detrusor hyperreflexia[73].

Recently, another exciting neurosurgical technique has been developed in which a new neuronal loop is created. Patients undergo a limited laminectomy between L4 and S2 and the nerve roots are exposed. The L5 ventral root is identified by electrostimulation producing plantar flexion of the foot and transected at the orifice. The S3 ventral root is transected near the cord. The proximal stump of the ventral root of L5 is then anastomosed to the distal stump of the S3 ventral root. This is performed unilaterally. The originators of this method first reported their successes in spinal cord injured adults, showing that via percutaneous electrical stimulation or scratching the skin in the L7 dermatome, a detrusor contraction could be initiated[74]. The follow-up data after three years showed that 10 of the 15 patients (67%) regained satisfactory bladder control, and residual urine decreased from an average of 332 to 31 milliliters. Overflow incontinence and urinary tract infection were no longer clinical issues for these patients[75].

Hence, this same group decided to apply this neurosurgical technique to the spina bifida population. The cohort consisted of 20 children, average age 11 years, and 17 (85%) attained increased bladder storage capacity and emptying functions, including the ability to sense fullness and to initiate bladder emptying with L5 dermatome stimulation, and after six months post-operatively, were dry. The negative of this trial was that 5 of the 17 children who had success also had signs of partial loss of L5 motor function. In the 3 patients who had no improvement, 2 were noted to have significant scarring in the spinal canal due to their previous surgeries and therefore had inadequate neural rootlet identification. These results are still very promising, demonstrating that surgical alteration of the sacral innervation of patients with spina bifida can have significant beneficial effects on bladder function[76]. Additional trials are now being undertaken.

Hopefully, further advances in neuromodulation therapies will occur as we gain greater understanding of neurourology and how abnormal innervation affects bladder development *in utero* and bladder function post-natally. If we can reestablish more normal nerve – muscle interaction via electrical or surgical means, then this should allow for more normal bladder function that can decrease or eliminate the need for additional medical and/or surgical therapy of the neurogenic bladder in patients with spina bifida.

## CONCLUSIONS

Certainly, the methodology of bladder management for spina bifida patients is changing. While the gold standard remains early medical management with pharmacotherapy (anticholinergic medication) and clean intermittent catheterization in an effort to maintain healthy bladder dynamics, with subsequent bladder augmentation in patients that fail medical management, there are children for whom this is clearly not enough. Hopefully in the near future, as we make greater strides and advancements in the areas of tissue engineering and neuromodulation, there will be additional more attractive forms of therapy that will allow patients with spina bifida to keep their kidneys safe, their clothing dry, and their quality of life strong.
